# Repositioning Perindopril for Mitigation of Methotrexate-Induced Hepatotoxicity in Rats

**DOI:** 10.3390/ph18030358

**Published:** 2025-03-02

**Authors:** Hanan Abdelmawgoud Atia, Hemat A. Elariny, Marwa H. Abdallah, Amany M. Khalifa, Remon S. Estfanous, Maaly A. Abd Elmaaboud, Ahmed M. Kabel

**Affiliations:** 1 Department of Pharmacology and Toxicology, College of Pharmacy, University of Ha’il, Ha’il 81442, Saudi Arabia; 2 Department of Pharmaceutics, College of Pharmacy, University of Ha’il, Ha’il 81442, Saudi Arabia; 3 Department of Pathology, College of Medicine, University of Ha’il, Ha’il 81442, Saudi Arabia; 4 Anatomy and Embryology Department, Faculty of Medicine, Tanta University, Tanta 31527, Egypt; 5 Department of Pharmacology, Faculty of Medicine, Tanta University, Tanta 31527, Egypt

**Keywords:** perindopril, methotrexate, hepatotoxicity, inflammatory cascade, HMGB1, rats

## Abstract

**Background/Objectives:** Methotrexate is a folate antagonist that has proven efficacy as an anticancer and immunomodulatory agent. However, the possible incidence of overt hepatotoxicity represents a challenge for its clinical use. Up till now, no single remedy has been considered an effective solution to this important adverse effect. Perindopril is an angiotensin-converting enzyme inhibitor that is widely used for the treatment of hypertension. Due to the involvement of the renin–angiotensin system in the pathogenesis of methotrexate-elicited hepatotoxicity, investigating the efficacy of perindopril in this condition may be of particular interest. The current work aimed at an evaluation of the potential effects of perindopril in a rat model of methotrexate-induced hepatotoxicity and tried to precisely determine the molecular mechanisms that may represent the basis of these effects. **Methods:** In a model of methotrexate-elicited hepatotoxicity in male Wistar rats, the effects of different doses of perindopril were evaluated at the level of the biochemical measurements and the morphological examination. **Results:** Oral administration of perindopril to methotrexate-injected rats exhibited a dose-dependent significant improvement in daily food intake; the restoration of the functions of hepatocytes; the potentiation of antioxidant defense mechanisms; the abrogation of the different signaling pathways involved in liver inflammation, apoptosis, and fibrosis; and an enhancement in AMPK/mTOR-driven autophagy when compared to animals that received only a methotrexate injection. These events were reflected in the morphological appearance of the different studied groups. **Conclusions:** This study presents perindopril as a promising remedy for mitigation of the hepatotoxic effects that occur as a consequence of treatment with methotrexate.

## 1. Introduction

Methotrexate is an antifolate agent that belongs to the antimetabolite family of drugs that has proven efficacy in the management of certain types of malignancies including leukemia and lymphoma [1]. In addition, its antiproliferative effects confer a unique ability to combat disease states characterized by rapid cell growth and proliferation such as severe psoriasis [2]. Moreover, methotrexate has been reported to modulate T-cell function, cytokine production, the expression of cellular adhesion molecules, and humoral responses, thus, representing an effective agent for the management of autoimmune diseases and rheumatoid arthritis [3]. Despite of these beneficial effects, the clinical use of methotrexate is limited by the increased incidence of adverse effects including gastrointestinal disturbances, headache, mouth ulcers, elevated serum transaminases, and renal insufficiency [4]. Of these, hepatotoxicity represents the most common and the most serious adverse effect of methotrexate [5]. The proposed mechanisms of this type of toxicity include inhibition of the key antioxidant enzymes in hepatic tissues such as cytosolic nicotinamide adenine dinucleotide phosphate (NADP)-dependent dehydrogenase and NADP malic enzyme together with the induction of direct hepatocyte injury, the enhancement of apoptosis, and the augmentation of inflammatory and fibrogenic mediators, with the net result of liver fibrosis and even overwhelming cirrhosis [6]. Although multiple research trials have been directed toward the amelioration of these toxic effects, none of these trials had proven efficacy in the safe prevention of these toxic events [7]. Thus, the search for possible agents that may combat the mechanistic pathways that are involved in the pathogenesis of methotrexate-induced hepatotoxicity without interference with the favorable effects of methotrexate is of particular interest.

The renin–angiotensin system plays a fundamental role in the regulation of blood volume, serum electrolytes, and blood pressure [8]. In addition, its expression in various organs, including the liver, kidney, and heart, has been proven to play a key role in the normal functioning of these organs [9]. In the liver, modulation of the renin–angiotensin system has been reported to significantly affect the physiological processes performed by the hepatocytes [10]. Angiotensin II, which is produced as a result of the activation of the renin–angiotensin system, was found to have a detrimental role in the pathogenesis of liver disease through exerting pro-oxidant, fibrogenic, and proinflammatory effects on hepatic tissues [11]. In addition, angiotensin II has been reported to have a modulatory effect on apoptotic signals, which in turn may significantly affect the survival rates of hepatocytes [12]. Interestingly, the inhibition of angiotensin II receptors was reported to significantly ameliorate the inflammatory changes in the hepatic tissues and mitigate the fibrotic changes frequently encountered in chronic liver diseases [13].

Perindopril is a member of the renin–angiotensin antagonists that is commonly used for the treatment of patients with elevated blood pressure [14]. Recent reports have proven that the dysregulation of the renin–angiotensin system may play a key role in the pathogenesis of methotrexate-induced cytotoxicity [15]. Patients having significantly increased levels of angiotensin II in the liver have shown an enhancement in the pro-oxidant and inflammatory changes in the hepatic tissues with subsequent detrimental effects on hepatic functions [16]. In addition, methotrexate, after entering the hepatic cell, is converted into methotrexate polyglutamate, which inhibits DNA synthesis and subsequently reduces AMPK levels, which is an inducer of SIRT1; the reduction in SIRT1 makes the liver cell vulnerable to oxidative stress, inflammation, and apoptosis. Perindopril could increase Ang (1–7), which turns on a hepatic protective RAS pathway involving the activation of AMPK and autophagy and combat inflammation and fibrosis [6,17,18]. These reports, in addition to the antioxidant, anti-inflammatory, and antiapoptotic properties of perindopril, may open new horizons for the amelioration of methotrexate-elicited hepatotoxicity [19].

Sirtuin 1 (SIRT1) is one of the histone deacetylases that is involved in the regulation of the different physiological processes in the body [20]. Recent reports have delineated the possible role played by SIRT1 in the regulation of the functions of the hepatocytes and abrogation of methotrexate-elicited hepatotoxic effects [21]. This role may be a consequence of its balance-keeping effect on the redox status in the hepatic tissues together with its ability to modulate the different signaling pathways involved in cellular inflammation and survival in the hepatic tissues [22]. Additionally, overexpression of SIRT1 in the hepatocytes was reported to promote the DNA repair mechanisms and enhance the autophagy signals in the hepatic tissues, thus, limiting the extent of methotrexate-mediated cellular damage and preventing further hepatic injury [23].

High-Mobility Group Box 1 (HMGB1) is a non-histone chromatin-associated protein that normally stabilizes nucleosome formation and plays a key role in genetic transcription [24]. Recent reports stated that HMGB1 may be incriminated in the pathogenesis of methotrexate-elicited hepatotoxicity [25]. This may be supported by the findings that it may act as a damage-associated molecular pattern molecule that signifies the presence of hepatocytes damage to the immune system [26]. In addition, HMGB1 was proven to interact with the receptors involved in the inflammatory cascade including the receptors for advanced glycation end products (RAGE) and toll-like receptors (TLRs) [27]. This consequently leads to the activation of the various signaling pathways that mediate inflammation, apoptosis, and fibrosis of the hepatic tissues, which are the critical events that are pathognomonic to methotrexate-elicited hepatotoxicity [28].

The current work represented a trial to investigate the effects of perindopril on a rat model of methotrexate-induced hepatotoxicity and to precisely determine the potential mechanisms that may contribute to these effects.

## 2. Results

### 2.1. Perindopril Dose-Dependently Combatted the Effect of Methotrexate on the Daily Food Intake

**Figure 1:**
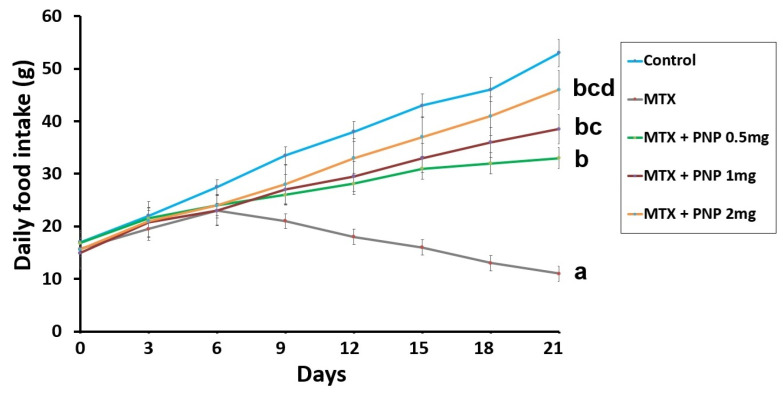
Effect of the different treatments on the daily food intake throughout the whole study (mean ± SD). ^a^ Significant compared to the control group; ^b^ Significant relative to the untreated methotrexate group; ^c^ Significant relative to methotrexate group treated with perindopril 0.5 mg/kg/day; ^d^ Significant relative to methotrexate group treated with perindopril 1 mg/kg/day. MTX: methotrexate; PNP: perindopril.

Animals treated with methotrexate alone exhibited a significant decline in the daily food intake relative to the control animals. Interestingly, the methotrexate-injected groups treated with perindopril showed dose-dependent improvement in the daily food intake relative to the group injected with methotrexate alone (Figure 1).

### 2.2. Perindopril Dose Dependently Ameliorated the Effect of Methotrexate on the Liver Function Tests

**Figure 2:**
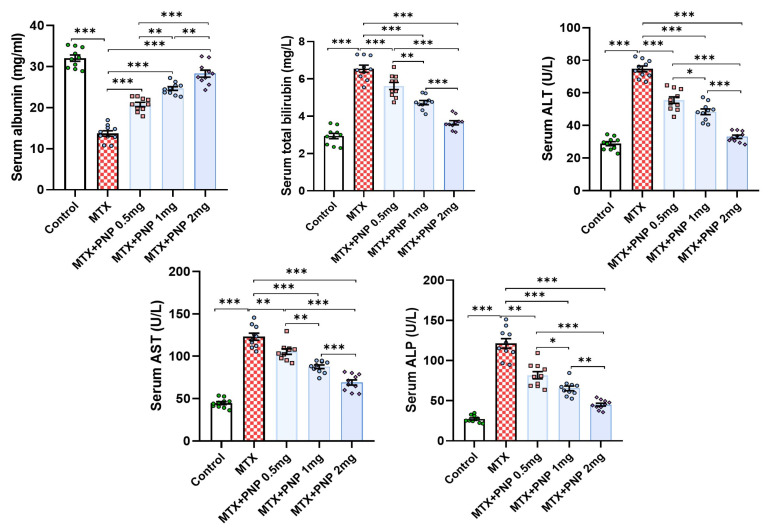
Effect of the different doses of perindopril on the liver function tests in the animal groups treated with methotrexate (mean ± SD); * = *p* < 0.05, ** = *p* < 0.01, *** = *p* < 0.001; MTX: methotrexate; PNP: perindopril; ALT: alanine transaminase; AST: aspartate transaminase; ALP: alkaline phosphatase.

Methotrexate injection elicited a significant perturbation in the liver functions as evidenced by a substantial decline in serum albumin levels associated with a significant increase in serum total bilirubin, ALT, AST, and ALP when compared to the values of the same parameters encountered with the control group. Interestingly, the methotrexate-injected groups treated with perindopril exhibited a significant improvement of the liver functions manifested by a significant increase in serum albumin associated with a significant decrement in the serum levels of total bilirubin, ALT, AST, and ALP when compared versus animals treated with methotrexate alone. This effect was apparent in a dose-dependent manner (Figure 2).

### 2.3. Perindopril Dose Dependently Mitigated the Effect of Methotrexate on the Redox Status of the Hepatic Tissues

**Figure 3:**
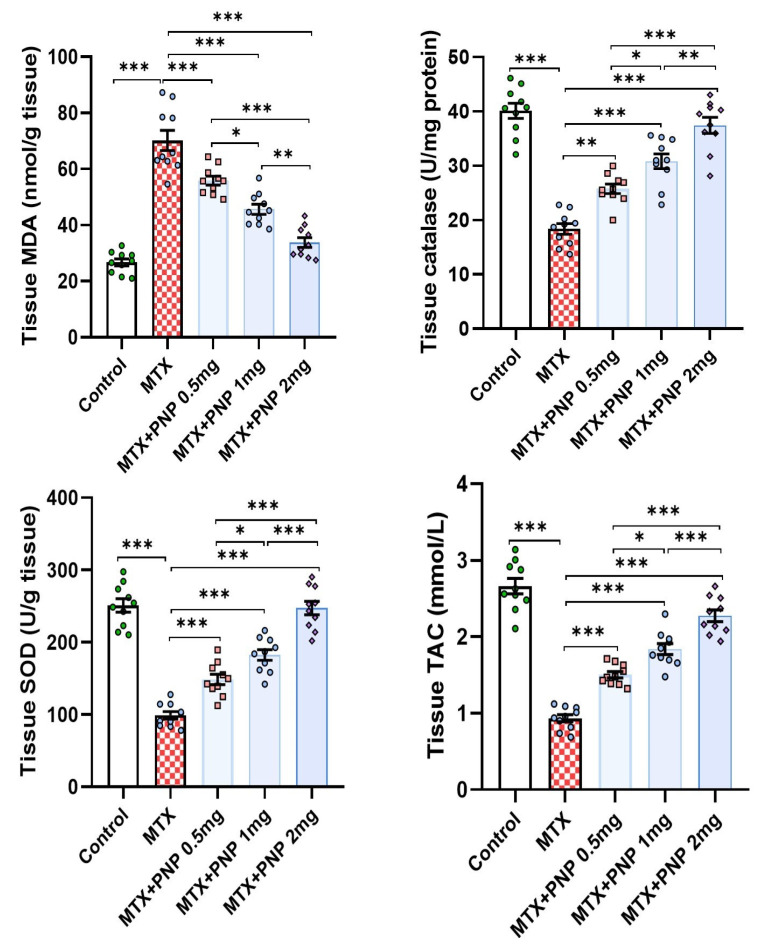
Effect of the different doses of perindopril on the redox status of the hepatic tissues of the animal groups treated with methotrexate (mean ± SD); * = *p* < 0.05, ** = *p* < 0.01, *** = *p* < 0.001; MTX: methotrexate; PNP: perindopril; MDA: malondialdehyde; SOD: superoxide dismutase; TAC: total antioxidant capacity.

As shown in Figure 3, methotrexate injection elicited a significant perturbation of the redox status of the hepatic tissues manifested by a significant elevation of MDA content of the hepatic tissues and a significant decrease in the hepatic tissue CAT, SOD, and TAC. Meanwhile, administration of perindopril to methotrexate-injected rats was able to restore the antioxidant defenses in the hepatic tissues manifested by a significant elevation of the tissue levels of CAT, SOD, and TAC and decremental decline in tissue MDA levels relative to rats treated with methotrexate alone. As concluded from the results, these admirable effects were dose-dependent.

### 2.4. Perindopril Dose Dependently Combatted the Effect of Methotrexate on SIRT1 and PPAR-γ Content of the Hepatic Tissues

**Figure 4:**
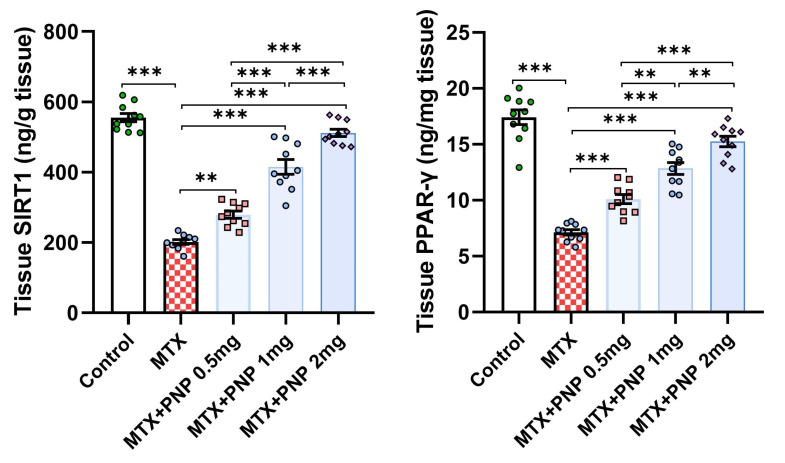
Effect of the different doses of perindopril on sirtuin-1 and peroxisome proliferator-activated receptor gamma levels in the hepatic tissues of the animal groups treated with methotrexate (mean ± SD); ** = *p* < 0.01, *** = *p* < 0.001; MTX: methotrexate; PNP: perindopril; SIRT1: sirtuin-1; PPAR-γ: peroxisome proliferator-activated receptor gamma.

Figure 4 demonstrates the effect of the different treatments on the hepatic tissue content of SIRT1 and PPAR-γ. Rats treated with only methotrexate exhibited a significant decrease in SIRT1 and PPAR-γ levels in the hepatic tissues relative to the control animals, which signifies the regulatory role of PPAR-γ on SIRT1 expression. Amazingly, perindopril administration to methotrexate-injected rats induced a dose-dependent significant elevation in the hepatic tissue SIRT1 and PPAR-γ levels when compared to the levels of the same parameters in the group that received methotrexate alone.

### 2.5. Perindopril Dose Dependently Abrogated the Effect of Methotrexate on KEAP1, Nrf2, and HO-1 Content of the Hepatic Tissues

**Figure 5:**
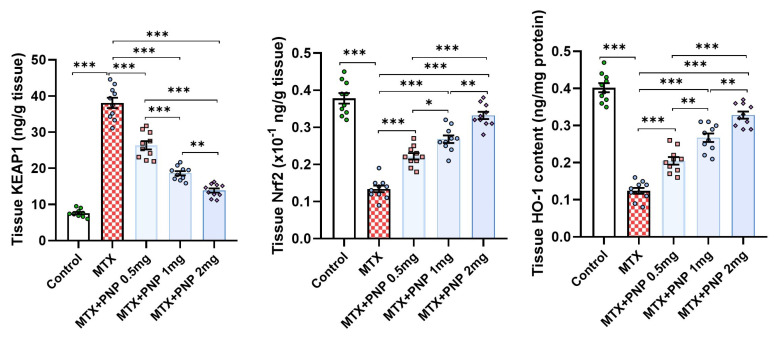
Effect of the different doses of perindopril on KEAP1, Nrf2, and HO-1 content of the hepatic tissues of the animal groups treated with methotrexate (mean ± SD); * = *p* < 0.05, ** = *p* < 0.01, *** = *p* < 0.001; MTX: methotrexate; PNP: perindopril; KEAP1: Kelch-like ECH-associated protein 1; Nrf2: nuclear factor erythroid 2-related factor 2; HO-1: heme oxygenase-1.

Figure 5 shows the effect of the different treatments on KEAP1/Nrf2/HO-1 signaling in the hepatic tissues. The significant increase in tissue KEAP1 levels associated with the significant decline in the hepatic tissue content of Nrf2 and HO-1 induced by methotrexate injection was dose-dependently mitigated with the administration of perindopril. This clarifies the role played by KEAP1 in the regulation of Nrf2/HO-1 signaling.

### 2.6. Perindopril Dose Dependently Decreased the Hepatic Tissue Levels of the Proinflammatory Cytokines in Rats Treated with Methotrexate

**Figure 6:**
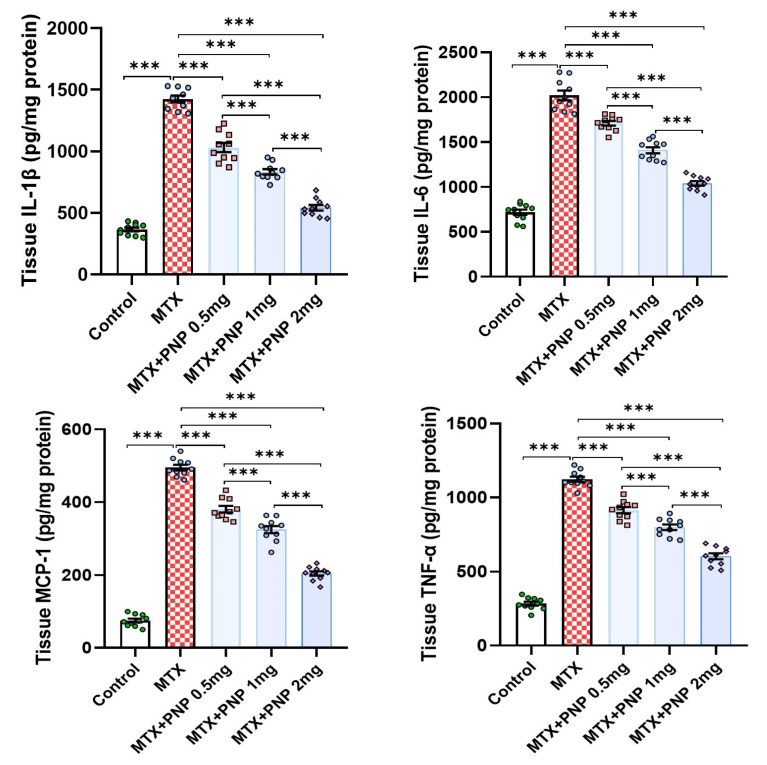
Effect of the different doses of perindopril on IL-1β, IL-6, MCP-1, and TNF-α levels in the hepatic tissues of the animal groups treated with methotrexate (mean ± SD); *** = *p* < 0.001; MTX: methotrexate; PNP: perindopril; IL-1β: interleukin 1-beta; MCP-1: monocyte chemoattractant protein 1; TNF-α: tumor necrosis factor-alpha.

As depicted in Figure 6, methotrexate injection induced a state of intense inflammatory response as evidenced by a significant elevation of the hepatic tissue levels of IL-1β, IL-6, MCP-1, and TNF-α when compared to the control values. This inflammatory response was significantly ameliorated with the administration of perindopril in a dose-dependent way that highlighted the potent anti-inflammatory effects of perindopril in the hepatic tissues.

### 2.7. Perindopril Dose Dependently Mitigated the HMGB1/RAGE/Nuclear Factor Kappa B (NF-κB) Axis in the Hepatic Tissues of Rats Treated with Methotrexate

**Figure 7:**
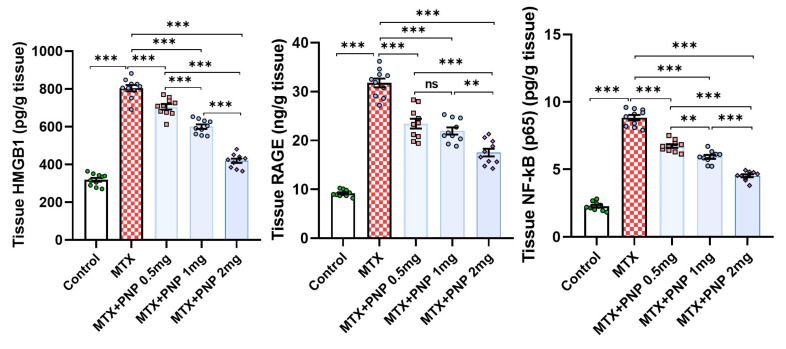
Effect of the different doses of perindopril on HMGB1/RAGE/NF-κB axis in the hepatic tissues of the animal groups treated with methotrexate (mean ± SD); ns = non-significant, ** = *p* < 0.01, *** = *p* < 0.001; MTX: methotrexate; PNP: perindopril; HMGB1: High-Mobility Group Box 1; RAGE: receptors for advanced glycation end products; NF-κB: nuclear factor kappa B.

Figure 7 gives an idea about the effect of different treatments on the HMGB1/RAGE/NF-κB axis in the hepatic tissues. The hepatic tissue specimens retrieved from rats treated with methotrexate alone had shown a significant increase in HMGB1, RAGE, and NF-κB p65 levels relative to the hepatic tissue specimens obtained from the control group. These events were dose-dependently reversed in the groups treated with perindopril, thereby signifying the combatting effect of perindopril against the effects of methotrexate on the HMGB1/RAGE/NF-κB axis in the hepatic tissues.

### 2.8. Perindopril Dose Dependently Reversed the Effect of Methotrexate on Phospho-mTOR, Total AMPK, and LC3-II in the Hepatic Tissues

**Figure 8:**
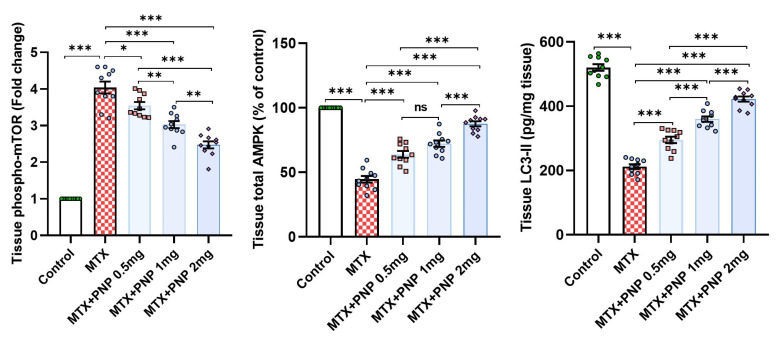
Effect of the different doses of perindopril on phospho-mTOR, total AMPK, and LC3-II levels in the hepatic tissues of the animal groups treated with methotrexate (mean ± SD); ns = non-significant, * = *p* < 0.05, ** = *p* < 0.01, *** = *p* < 0.001; MTX: methotrexate; PNP: perindopril; phospho-mTOR: phosphorylated mammalian target of rapamycin; AMPK: adenosine monophosphate-activated protein kinase; LC3-II: microtubule-associated protein light chain 3.

Compared to the control group, methotrexate in the present work demonstrated an ability to inhibit the autophagic signals in the hepatic tissues, as evidenced by a significant elevation of the hepatic tissue content of phospho-mTOR (Ser2448) and a significant decline of the total AMPK and LC3-II levels in the hepatic tissues. These changes were dose-dependently abrogated with the administration of perindopril, which uncovered its potential autophagy-inducing effects (Figure 8).

### 2.9. Perindopril Dose Dependently Abrogated the Fibrogenic Process Induced by Methotrexate in the Hepatic Tissues

**Figure 9:**
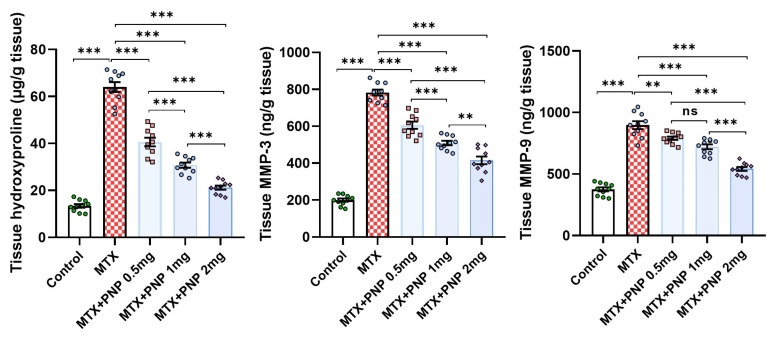
Effect of the different doses of perindopril on hydroxyproline, MMP-3, and MMP-9 in the hepatic tissues of the animal groups treated with methotrexate (mean ± SD); ns = non-significant, ** = *p* < 0.01, *** = *p* < 0.001; MTX: methotrexate; PNP: perindopril; MMP: matrix metalloproteinase.

Figure 9 demonstrates the effect of the different treatments on the fibrogenic signals in the hepatic tissues. As compared to the control animals, the group treated with methotrexate alone had shown strong evidence of hepatic fibrosis as proven by the significant increase in the hepatic tissue content of hydroxyproline, MMP-3, and MMP-9. Amazingly, all groups treated with perindopril exhibited a significant mitigation of the aforementioned fibrogenic signals in the hepatic tissues, an effect that appeared to be largely dose-dependent.

### 2.10. The Changes Elicited by Administration of Methotrexate With or Without the Different Doses of Perindopril on the Histopathological View of the Hepatic Tissues

**Figure 10:**
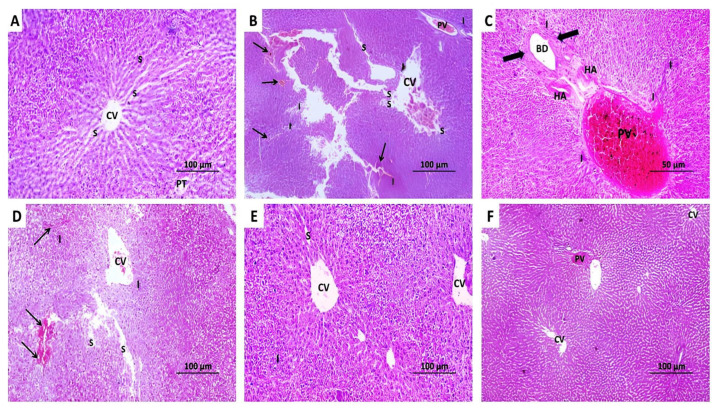
Hematoxylin and eosin-stained sections of the liver from (**A**) the control group showing the characteristic hexagonal classic hepatic lobules with central veins (CV) at the center and portal tracts (PT) at the periphery. The polygonal hepatocytes are arranged in cords separated by blood sinusoids (S) (×100); (**B**) methotrexate group exhibiting loss of normal hepatic architecture with dilated and markedly congested central vein (CV) and portal venules (PV) with diffuse perivascular, periportal, and interstitial inflammatory cellular infiltration (I). The central parts of the blood sinusoids (S) appear dilated in some regions with focal areas of hepatic necrosis (Thin arrows) (×100); (**C**) Portal tract of methotrexate group showing dilated congested portal venules (PV) and hepatic arterioles (HA) with proliferation of bile ductules (BD) (Thick arrows). Also, scattered areas of inflammatory cellular infiltration (I) are seen in the portal area (×400); (**D**) methotrexate group treated with a small dose of perindopril revealing a significant improvement in the hepatic architecture with cords of normal hepatocytes that surround a mildly dilated central vein (CV). Some hepatic sinusoids appear mildly dilated (S) with scanty areas of hepatic necrosis (Thin arrows) and inflammatory cellular infiltration (I) (×100); (**E**) methotrexate group treated with a moderate dose of perindopril showing minimal dilatation of the central veins which are surrounded by cords of hepatocytes with acidophilic cytoplasm and vesicular nuclei. Some of the blood sinusoids (S) appear dilated with minimal inflammatory cellular infiltration (I) (×100); (**F**) methotrexate group treated with a large dose of perindopril exhibiting restoration of the normal hepatic histomorphic structure with appearance of the classic hexagonal hepatic lobules with apparently normal central veins (CV) and portal tracts with minimal congestion of the portal venules (PV) (×100).

In the present study, the control rats showed the characteristic hexagonal classic hepatic lobules with central veins at the center, portal tracts at the periphery, and polygonal hepatocytes arranged in cords separated by blood sinusoids (Figure 10A). The administration of methotrexate elicited a significant loss of the normal hepatic architecture with dilated and markedly congested central veins and portal venules with diffuse inflammatory cellular infiltration. The central parts of the blood sinusoids became dilated with focal areas of hepatic necrosis (Figure 10B). The portal tracts of the group that received methotrexate alone showed dilated congested portal venules and hepatic arterioles with proliferation of bile ductules and diffuse inflammatory cellular infiltration (Figure 10C). These changes were dose-dependently ameliorated with perindopril administration manifested by a significant improvement in the hepatic architecture with mild dilatation of the central veins, the portal venules, and the hepatic sinusoids with a significant decline in the extent of hepatic necrosis and the inflammatory cellular infiltration (Figure 10D–F).

### 2.11. The Impact of Administration of Methotrexate With or Without the Different Doses of Perindopril on the Extent of He Immunohistochemical Positive Expression of Cleaved Caspase 3 in the Hepatic Tissue Specimens

**Figure 11:**
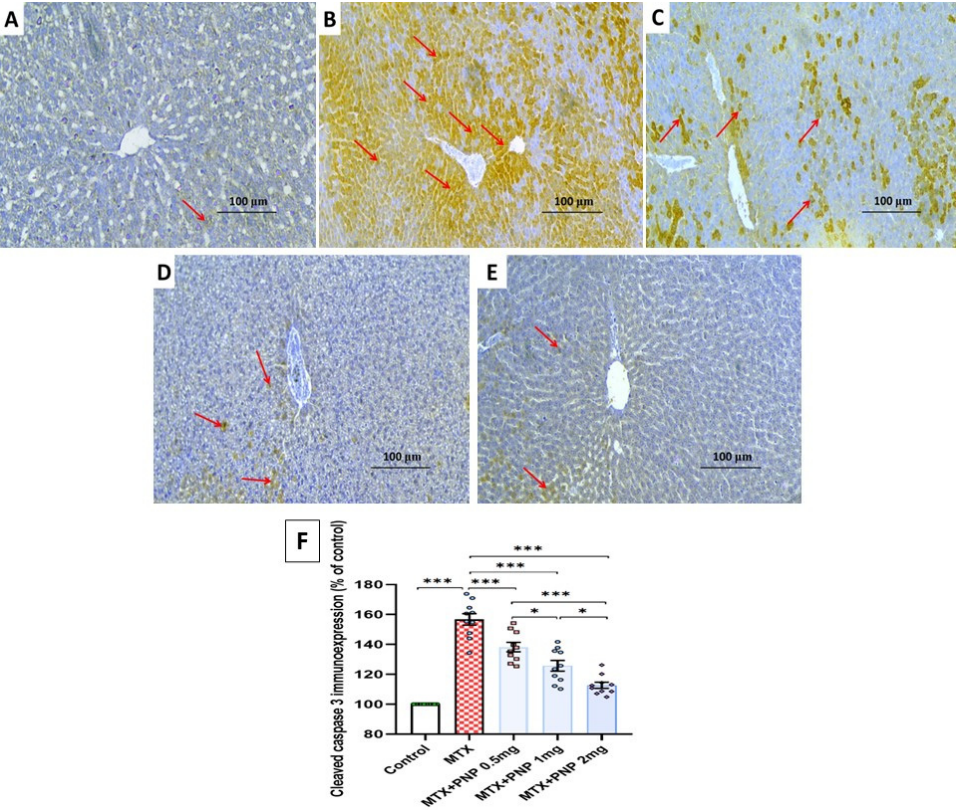
Hepatic tissue sections of immunohistochemical staining of cleaved caspase 3 in (**A**) The control group clarifying minimal positive immunostaining for cleaved caspase 3; (**B**) The group that received methotrexate alone exhibiting strongly positive immunostaining for cleaved caspase 3; (**C**–**E**) Methotrexate-injected groups treated with small, moderate, and large doses of perindopril, respectively, showing mild positive immunostaining for cleaved caspase 3; (**F**) Quantitative representation of the percentage of cleaved caspase 3 immune expression in the different studied groups (% of the control); * = *p* < 0.05, *** = *p* < 0.001; MTX: methotrexate; PNP: perindopril.

The hepatic tissue sections of animals injected with methotrexate alone revealed a significant enhancement of the immune expression of cleaved caspase 3 when compared to the control animals (Figure 11A,B,F). Tissue cleaved caspase 3 immuno-expression was dose-dependently mitigated in the livers of the groups treated with the different doses of perindopril (Figure 11C–F), which gave a clear indication of its antiapoptotic effects.

### 2.12. Perindopril Dose-Dependently Combatted Methotrexate-Elicited Perturbations in the Electron Microscopic Picture of the Hepatic Tissues

**Figure 12:**
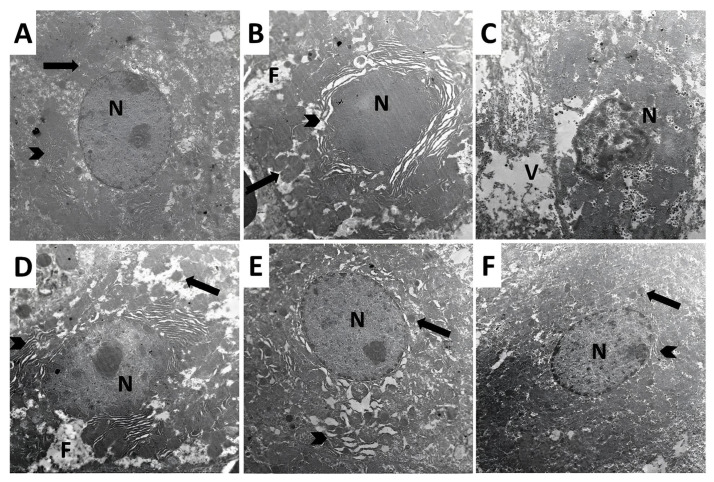
Electron micrographs of ultrathin sections in the liver from animals of (**A**) the control group showing normal architecture of the hepatic tissues. The nuclei (N) appeared spherical with regular outlines with a small amount of heterochromatin in the peripheral regions and a large central amount of euchromatin and prominent nucleolus. The cytoplasm of the hepatocytes contains abundant mitochondria (Arrow) with well-developed cristae and the rough endoplasmic reticulum (RER) consists of closely packed parallel and flattened cisternae (Arrowhead); (**B**,**C**) methotrexate-treated group showing shrunken irregular nucleus with dispersed chromatin (N) and reduced number of the mitochondria with disrupted cristae (Arrow). The cisternae of the rough endoplasmic reticulum (RER) of the hepatocytes are fragmented (Arrowhead) with extensive fat droplets (F) and marked cytoplasmic vacuolation (V); (**D**) methotrexate group treated with a small dose of perindopril revealing irregular nucleus with preserved nucleolus (N). There is a mild increase in the number of the viable mitochondria with mild disrupted cristae (Arrow), partly preserved cisternae of the rough endoplasmic reticulum (Arrowhead), and small number of fat droplets could be observed (F); (**E**) methotrexate group treated with a moderate dose of perindopril exhibiting a spherical nucleus with regular wall and preserved nucleolus (N). There is moderate increase in the number of the mitochondria with mild disrupted cristae (Arrow) with mild disruption and wide separation of the cisternae of the rough endoplasmic reticulum (Arrowhead); (**F**) methotrexate group treated with a large dose of perindopril showing a normal spherical nucleus with intact regular walls and preserved nucleolus (N). The mitochondria are abundant with preserved cristae (Arrow) and the rough endoplasmic reticulum cisternae appear nearly normal with mild dilatation (Arrowhead).

The control group showed a normal appearance of the hepatic tissue architecture with spherical nuclei showing regular outlines, a small amount of heterochromatin at the peripheral regions, a large central amount of euchromatin, and prominent nucleoli. The cytoplasm of the hepatocytes showed abundant mitochondria and rough endoplasmic reticulum (RER) with preserved cisternae (Figure 12A). The group treated with methotrexate alone exhibited shrunken irregular nuclei with dispersed chromatin, a decreased number of viable mitochondria with disrupted cristae, fragmentation of the cisternae of RER, extensive fat droplets, and marked cytoplasmic vacuolations (Figure 12B,C). Treatment with perindopril dose-dependently counteracted the effects of methotrexate on the electron microscopic picture of the hepatic tissues with a significant decrease in the irregularity of the nuclei with preserved nucleoli, dose-dependent increase in the number of the viable mitochondria with partly preserved cisternae of RER, and dose-dependent decline in the number of fat droplets (Figure 12D–F).

## 3. Discussion

Methotrexate is an antimetabolite agent that has proven efficacy in the management protocols of malignant conditions, autoimmune disorders, and in the induction of therapeutic abortion [29]. Its efficacy in the aforementioned conditions is attributed to its ability to produce competitive inhibition of dihydrofolate reductase enzyme, thereby inhibiting the conversion of dihydrofolate to the active tetrahydrofolate with the net result of interference with DNA synthesis [30]. Owing to its selectivity on the S-phase of the cell cycle, it is particularly effective against rapidly dividing cells, thereby inhibiting the growth and proliferation of these cell types, which may, therefore, cause a wide range of adverse effects [31]. Among these unwanted effects, the possible induction of hepatotoxicity represents an obstacle that may significantly interfere with the efficacy of methotrexate [1]. The incriminated mechanisms in the pathogenesis of methotrexate-elicited hepatotoxicity include perturbation of the redox state, augmentation of the inflammatory signals, and induction of apoptosis in the hepatic tissues [6]. This was evident in the present work where methotrexate administration elicited a significant decrease in the daily food intake associated with significant perturbation of the liver function tests manifested by increased liver enzymes ALT, AST, and ALP. In addition, increased bilirubin and a reduction in albumin were associated with a significant derangement of the hepatic histopathological and electron microscopic pictures relative to the control group. The elevation of the liver enzymes is due to their release from the damaged liver cells. Although this would indicate hepatic injury, they still lack specificity due to their presence in the cardiac and muscular tissues. The elevation of liver enzymes in the current study was associated with increased serum bilirubin levels more than twice the control values and reduction in serum albumin, which comprise biological and clinical criteria of drug-induced liver injury in addition to deregulation of the tissue cytokines. This was confirmed by the histopathological and the electron microscopic changes showing derangement of liver cell nuclei, indicating that the methotrexate-induced hepatotoxicity in the present work was successfully supported by the biochemical, biological, and histopathological findings [32].

Recent reports highlighted the crucial role played by oxidative stress in methotrexate-induced hepatotoxicity [33]. Methotrexate was proven to enhance the excessive production of reactive oxygen species (ROS) with subsequent damage to the cellular and subcellular components including lipids, proteins, and DNA [34]. This in turn leads to overwhelming interference with the hepatic antioxidant defense mechanisms leading to the deleterious effects of oxidative stress including increased lipid peroxidation and DNA damage [35]. As a consequence, this triggers a series of inflammatory responses that exacerbate hepatic injury with the result of massive hepatic fibrosis and impairment of liver functions [36]. This was evident in the current work where rats treated with methotrexate alone exhibited a significant increase in hepatic tissue MDA associated with a significant decline in the antioxidant defenses relative to the control group. As a result, there was a significant elevation in the tissue proinflammatory cytokines, hydroxyproline, MMP-3, and MMP-9 levels relative to the control group, which highlighted the inflammatory responses and fibrosis as direct consequences of methotrexate-induced oxidative stress in the liver tissues.

In the current study, a dose-dependent amelioration of oxidative stress, decrement in the inflammatory reactions, and mitigation of the fibrogenic markers were observed in the animal groups treated with perindopril. This coincided with Gong et al. [37] who reported that the ability of perindopril to inhibit ROS production with its secondary events is the key contributor to its protective effects in the hepatic tissues. In addition, the production of angiotensin II, which is known to promote oxidative stress and fibrosis in various body organs including the liver, is significantly inhibited with angiotensin-converting enzyme inhibitors such as perindopril [38]. Moreover, perindopril was proven to augment the activity of antioxidant enzymes, which neutralize the lipid peroxidation mediators such as MDA, thereby restoring the oxidant/antioxidant balance in the hepatic tissues. As a consequence, perindopril prevents the activation of the hepatic stellate cells, which are the key regulators of collagen deposition and liver fibrosis [39].

In the present study, methotrexate injection induced a significant elevation of hepatic tissue KEAP1 levels associated with a significant decline in the hepatic tissue content of Nrf2 and HO-1 relative to the control animals. This was in agreement with the recent reports that highlighted the role of the KEAP1/Nrf2/HO-1 signaling pathway in the pathogenesis of methotrexate-induced hepatotoxicity [40]. KEAP1 is a regulatory protein that was reported to inhibit the Nrf2/HO-1 axis in different body tissues including the liver [41]. Methotrexate-enhanced KEAP1 overproduction may deprive the tissues of the antioxidant and the cytoprotective effects of Nrf2. Also, this interferes with the antioxidant and anti-inflammatory capacity of HO-1, an enzyme that degrades heme into biliverdin, carbon monoxide, and free iron [42]. Amazingly, perindopril in the present work was able to reverse these changes in a dose-dependent manner with repression of KEAP1 production and enhancement of the Nrf2/HO-1 axis in the hepatic tissues relative to the animals treated with methotrexate alone. This may be due to the ability of perindopril to enhance the activation and the translocation of Nrf2 into the nucleus, which then binds to the antioxidant response elements in DNA with subsequent increase in the expression of the various genes involved in the antioxidant and the anti-inflammatory defenses including the HO-1 gene [43]. Additionally, perindopril disrupts the interaction between KEAP1 and Nrf2, thus, protecting Nrf2 from KEAP1-mediated degradation [44].

The SIRT1/PPAR-γ axis is thought to play a key role in the protection of the different body organs against the toxic effects of methotrexate [45]. SIRT1 is a protein that has a pleiotropic regulatory effect on a wide range of cellular processes, including aging, inflammation, apoptosis, and oxidative stress [46]. Once activated, it enhances the antioxidant pathways and interferes with the inflammatory signals, thereby protecting the liver against oxidative stress and inflammation [47]. PPAR-γ is a nuclear receptor that modulates the expression of genes involved in lipid metabolism, glucose homeostasis, and anti-inflammatory responses [48]. SIRT1 was reported to deacetylate and enhance the activity of PPAR-γ with subsequent mitigation of oxidative stress and inflammation in the liver [49]. This was confirmed with the results of the present study where a significant decline in SIRT1 and PPAR-γ levels was noticed in rats treated with methotrexate alone when compared to the control values. Interestingly, perindopril in the present study exhibited a dose-dependent elevation in SIRT1 and PPAR-γ levels when compared to rats treated with methotrexate alone. This may be explained by the ability of perindopril to enhance the activity of SIRT1, which in turn deacetylates and activates several transcriptional factors and co-regulators including PPAR-γ with the result of improvement of the metabolic functions and reduction in inflammation [50].

In recent years, an emerging role for the HMGB1/RAGE/NF-κB pathway in the pathogenic sequences of methotrexate-induced hepatotoxicity was discovered [51]. HMGB1 is a protein that has been proven to be released from the damaged liver cells. Once released, it binds to RAGE on the outer surface of the hepatocytes which acts as a scaffold that triggers the inflammatory responses [52]. The most important of these responses is activation of NF-κB which is the key transcriptional factor that regulates the expression of the proinflammatory cytokines with subsequent hepatocellular damage [53]. This was evident in the present study where methotrexate-injected animals exhibited a significant increase in the hepatic tissue levels of HMGB1, RAGE, and NF-κB p65, which was reflected in a significant increase in the hepatic tissue proinflammatory cytokines and massive inflammatory cellular infiltration of the hepatic tissue specimens relative to the control animals. Coinciding with the findings of our study, Shen et al. [54] reported that perindopril reduces the expression of HMGB1 and RAGE with the net result of interference with the activity of NF-κB and its downstream signals. This subsequently produces a detrimental decline in the expression of the proinflammatory cytokines in the hepatic tissues with the net result of mitigation of the inflammatory and the fibrotic changes in the hepatic tissues [55].

The AMPK/mTOR signaling pathway is one of the key determinants of autophagy, which plays a fundamental role in the pathogenic events that are peculiar to methotrexate-induced cytotoxicity [56]. AMPK is an enzyme that plays a key role in energy production that activates autophagy by which the different cells undergo degradation and recycling to maintain energy balance and remove the damaged organelles [57]. When AMPK is activated, it enhances autophagy by inhibiting mTOR expression [58]. As demonstrated in the present study, animals injected with methotrexate exhibited a significant increase in phospho-mTOR levels in the hepatic tissues with a significant decline in tissue AMPK levels. These findings harmed autophagy in the hepatic tissues manifested by a significant decline in tissue LC3-II content, which is an important autophagy marker. Interestingly, these changes were dose-dependently reversed with the administration of perindopril, which highlighted its autophagy-facilitating properties in the hepatic tissues. These effects were attributed, at least in part, to its ability to improve overall cardiovascular health and to combat the deleterious consequences of oxidative stress in the hepatic tissues, thereby positively affecting liver functions [59].

The ongoing research efforts are directed toward the exploration of the role played by apoptosis in the pathogenic events of methotrexate-induced hepatotoxicity [60]. Methotrexate was found to cause ROS-mediated damage to the hepatocytes, which in turn enhances the activity of the apoptotic signals in the hepatic tissues. Of these, the caspases group of enzymes was proven to play a fundamental role [61]. As a consequence, the damaged cells were found to release certain mediators that attract the inflammatory cells to the damaged hepatic tissues, which can exacerbate the liver damage and the inflammatory responses [62]. This coincided with the immunohistochemical findings in the current study where the methotrexate-injected group exhibited a significant enhancement of the immune expression of cleaved caspase 3 relative to the control animals. Amazingly, perindopril usage in the present study was able to dose-dependently reduce this expression to approximate the reference control values. This may be attributed to the ability of perindopril to affect the rate of production of ROS, thereby protecting the hepatocytes against oxidative damage and apoptosis [63]. In addition, the inhibitory effect of perindopril on NF-κB expression deprives the apoptotic pathways of an important triggering agent [64]. Moreover, perindopril was proven to influence both the key pro-apoptotic factors such as caspase 3, and the antiapoptotic molecules such as Bcl-2, an effect that appears to be in favor of mitigation of apoptosis in the hepatic tissues [55].

In the current study, methotrexate induced a significant perturbation of the normal hepatic architecture with a significant vascular congestion and massive inflammatory cellular infiltration associated with focal hepatic necrosis and proliferation of the biliary ductules. These changes were associated with a significant enhancement of the hepatic tissue expression of cleaved caspase 3, the appearance of shrunken irregular nuclei with dispersed chromatin, a significant decline in the number and viability of the mitochondria, and diffuse cytoplasmic vacuolations. This was in the same line with Ali et al. [65] and Elsawy et al. [66] who attributed these changes to the direct toxic effect of methotrexate on the hepatic tissues associated with intense inflammatory response, induction of apoptosis, and interference with the mitochondrial functions. Interestingly, the findings of the present study showed that these changes were dose-dependently mitigated with perindopril administration manifested by a significant improvement in the hepatic architecture, a significant decline in tissue cleaved caspase 3 immune expression, and restoration of the normal structure of the nuclei, cytoplasm, and mitochondria. These results coincided with the findings of Betto et al. [67], Eid and El-Shitany [68], and Xu et al. [69] who reported that the antioxidant, anti-inflammatory, and antiapoptotic properties of ACEIs including perindopril may preserve the health status of the hepatocytes and offer a significant protection against various hepatotoxic agents.

Regarding the safety profile of perindopril in chronic administration, most of ACEIs are associated with instances of apparent cholestatic liver injury with mild elevation of liver enzymes, which was found to be self-limited and rare. Among the different ACEIs, perindopril is associated with the lowest rate of this adverse effect on the short-term scale that does not require dose modification, and it appears that it is an idiosyncratic reaction to a minor metabolite [70].

**Figure 13:**
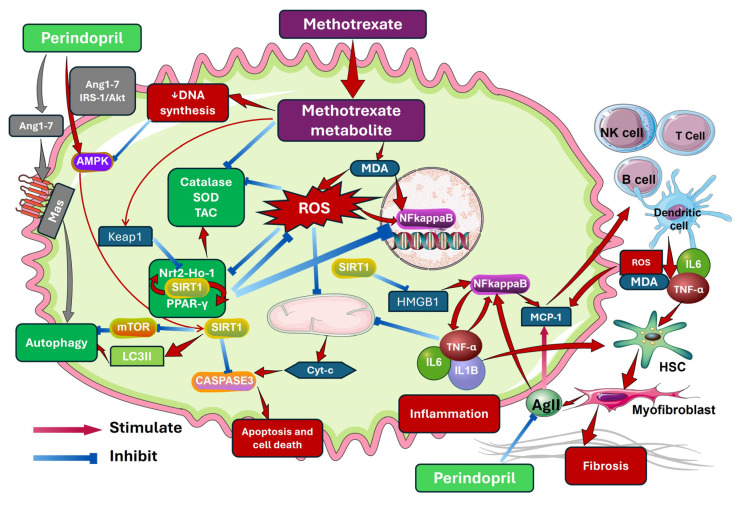
The mechanisms by which perindopril mitigates methotrexate-induced hepatotoxicity (This artwork was constructed using Reactome icon library and Smart Art Servier items).

Figure 13 represents the cross-talk and the interconnections between the different measured molecules in the current study. The mechanism of action of methotrexate was proposed as follows: methotrexate entering the hepatocyte is transformed into methotrexate metabolite that induces lipid peroxidation and elevated MDA, which enhances ROS production with consequent activation of NF-κB translocation into the nucleus and provocation of the proinflammatory cytokines such as IL-1β, TNF-α, IL-6 that creates an inflammatory environment, which activates the quiescent hepatic stellate cells to be transformed to myofibroblasts that secrete excessive collagen resulting in fibrosis. In addition, these myofibroblasts represent a source of angiotensin II that enhances ROS, NF-κB, and MCP-1, which in turn recruit immune cells to secrete more inflammatory cytokines and exacerbate the fibrogenesis process. Moreover, accumulation of the metabolites of methotrexate and ROS overproduction resulted in exhaustion and depletion of the antioxidant defenses of the hepatocytes with subsequent mitochondrial dysfunction and finally activation of caspase 3 and the apoptotic process. Furthermore, the resultant ROS depletes SIRT1, Nrf2, and PPAR-γ. The SIRT1 protein is vital for inhibiting ROS, NF-κB, and HMGB1, which promote inflammation. In addition, it has an inhibitory effect on caspase 3 and mTOR and enhances LC3-II, thereby playing a key role in the regulation of the processes of apoptosis and autophagy, respectively. Meanwhile, the metabolites of methotrexate inhibit DNA synthesis, which results in a reduction in AMPK with a subsequent reduction in SIRT1 activity. Perindopril would have protected the hepatocytes in the current study through the reduction in angiotensin II, which promotes the inflammatory and fibrogenic signals and oxidative stress in the hepatic tissues. Also, perindopril enhances AMPK, which induces SIRT1 with its protective mechanisms mentioned above [5,17,18].

The limitations of the current study include the absence of assessment of local RAS molecules inside the liver cells, such as measuring Ang (1–7) level and studying the connection between it and IRS-1/Akt that activate AMPK. In addition, the agonistic activity of perindopril via Ang (1–7) on Mas receptors in the liver cells and the linkage to the autophagy system need further research. Also, investigating the efficacy of perindopril on methotrexate-induced hepatotoxicity models with different dosages and duration of methotrexate application, and the effects of the studied drugs on the gut microbiota and amino acid metabolic pathways should be performed. Moreover, studying the perindopril effects on methotrexate induced hepatotoxicity in an animal model of cancer should be carried out to investigate if there is interference of perindopril with the different pathways that regulate cancer biology to be translatable to humans.

## 4. Materials and Methods

### 4.1. Ethical Considerations

The experimental protocol and procedures of the current study followed the ARRIVE guidelines and agreed with the U.K. Animals (Scientific Procedures) Act, 1986 and associated guidelines, EU Directive 2010/63/EU for animal experiments. The experimental protocol had official approval from the Research Ethics Committee of the Faculty of Medicine, Tanta University, Egypt (Approval code 36264PR882/10/24; Date of approval 7 October 2024).

### 4.2. Drugs and Reagents Used

Methotrexate was purchased in a powder form from LGC Standards, Teddington, Middlesex, TW11 0LY, UK (Product Code DRE-C15056900; CAS No. 59-05-2). Perindopril powder was supplied by Santa Cruz Biotechnology, Inc., Dallas, TX, USA (Catalog # sc-205799A; CAS No. 82834-16-0). Methotrexate powder was dissolved in 0.9% sodium chloride solution. Dimethyl sulfoxide (DMSO) 10% solution obtained from Hefei Home Sunshine Pharmaceutical Technology Co., Ltd., Hefei City, China (EINECS No. 200-664-3, CAS No. 67-68-5) was utilized for dissolution of perindopril. The remaining chemical compounds and reagents used in the current study were of analytical grade and were purchased from El Goumhoria Trade Pharmaceuticals and Chemicals and Medical Supplies Co., Tanta, Egypt.

### 4.3. Grouping of the Experimental Animals and the Study Design

**Figure 14:**
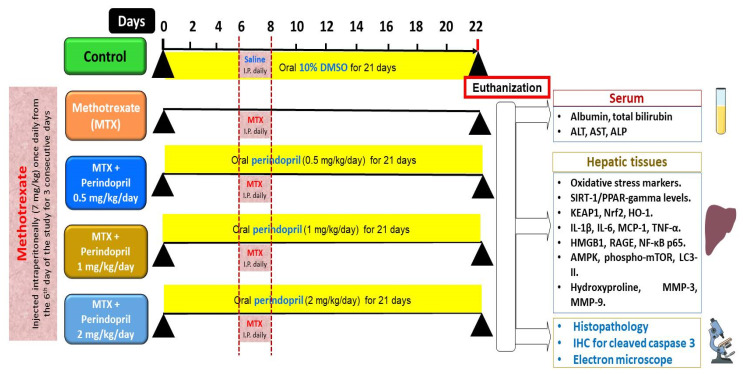
A representative diagram of the experimental protocol of the present study.

Fifty sexually mature male Wistar rats (8–10 weeks old, body weight 200–240 g) purchased from the animal house of the tumor biology department, National Cancer Institute, Cairo, Egypt were put in mesh wire cages for an acclimatization period of ten days during which they had free access to food and water with 12 h/12 h dark/light cycle. After that, these rats were randomly assigned to five equal groups of ten rats per group as illustrated in Figure 14 as follows: the control animals, which were intraperitoneally injected with 0.9% sodium chloride solution once daily for 3 consecutive days starting from the 6th day of the study concomitantly with daily administration of 10% DMSO solution by a gastric tube starting from day 0 of the study for 21 days; methotrexate group in which the animals were injected intraperitoneally with methotrexate (7 mg/kg) once daily for 3 consecutive days starting from the 6th day of the study [71]; methotrexate + perindopril small dose group in which the animals injected with methotrexate as described above received a daily oral dose of perindopril (0.5 mg/kg) [72]; methotrexate + perindopril moderate dose group in which rats injected with methotrexate as described above received a daily oral dose of perindopril (1 mg/kg) [72]; methotrexate + perindopril large dose group in which rats injected with methotrexate as described above received a daily oral dose of perindopril (2 mg/kg) [73]. Perindopril was administered once daily by gastric tube starting from day 0 of the study for 21 days.

### 4.4. Determination of the Daily Food Intake in the Different Studied Groups

Every 3 days throughout the study, the presented food to animals was pre-weighed, and 24 h later, the animals were removed from their cages. Then, the remaining food and the spilled-out food particles were weighed and recorded. The daily food intake was calculated by subtracting the weight of the remaining food and the spilled-out food particles from the weight of the food presented to the animals as previously described by Krízova et al. [74].

### 4.5. Collection of the Blood and the Liver Tissue Samples from the Different Animal Groups

On the 22nd day of the study, all animals were fasted overnight and anesthetized with thiopental sodium (30 mg/kg body weight, intraperitoneal) [75]. Then, the blood samples were withdrawn from the retro-orbital plexus, centrifuged at 3000 rpm for 20 min, and the separated sera were utilized for the assessment of the liver function tests. Then, rats from all groups were euthanized by cervical dislocation, and the liver tissue samples were excised. Parts of these tissues were further processed for histopathological and immunohistopathological evaluation and other parts were homogenized using an ultrasonic homogenizer. The yielded homogenate was further centrifuged using a cooling centrifuge, and the resulting supernatant represented the working solution in which the different tissue biochemical parameters were assessed.

### 4.6. Determination of the Serum Biochemical Parameters

Crystal Chem, Elk Grove Village, IL 60007, USA was the source of ELISA kits used for the quantification of serum albumin levels (catalog # 80662). Serum total bilirubin was assessed by kits purchased from BlueGene Biotech, Shanghai, China (catalog # E02T0143). Spectrophotometric determination of serum levels of alanine transaminase (ALT) and aspartate transaminase (AST) was carried out using colorimetric kits supplied by BioDiagnostics, Cairo, Egypt (catalog # MD 252 and MD 253, respectively). Tianjin AVIVA Systems Biology, Tianjin, 300457, China, was the provider of kits used for the assay of serum alkaline phosphatase (ALP) (catalog # OKEH03286). The steps used for the quantification of the aforementioned parameters in serum followed the manufacturer’s instructions.

### 4.7. Assessment of the Biochemical Measurements in the Supernatant of the Hepatic Tissues’ Homogenates

#### 4.7.1. Evaluation of the Redox Status of the Hepatic Tissues

The extent of lipid peroxidation in the hepatic tissues was assessed by measuring the malondialdehyde (MDA) content of the supernatant of the hepatic tissue homogenate. This was performed using a spectrophotometric assay, where MDA reacts with thiobarbituric acid at 95 °C in an acidic environment, resulting in a pink color. The absorbance of this color was then measured at a wavelength of 532 nm [76]. 

The supernatant of the centrifuged hepatic tissue homogenate was utilized for the assessment of catalase (CAT) activity by kits supplied by RayBiotech, Inc., Peachtree Corners, GA 30092, USA (catalog # MA-CAT-1). Before the assessment, the samples were treated with triton X-100 to solubilize the enzyme. This assay depends on spectrophotometric detection of the rate of disappearance of hydrogen peroxide, which was taken as an index of CAT activity, which was expressed as units/mg protein [77]. Also, the activity of superoxide dismutase (SOD) in the hepatic tissues was measured spectrophotometrically at a wavelength of 440–460 nm using kits obtained from BioDiagnostics, Cairo, Egypt (catalog # SD 25 21), according to the vendor’s protocol.

The total antioxidant capacity (TAC) of the supernatant of the centrifuged hepatic tissue homogenate was assayed using kits purchased from AFG Bioscience, Northbrook, Illinois, 60062, USA (catalog # EK720634) according to the provider’s instructions.

#### 4.7.2. Quantification of the Hepatic Tissue Content of Sirtuin-1 (SIRT1) and Peroxisome Proliferator-Activated Receptor Gamma (PPAR-γ)

Kits purchased from MyBioSource, San Diego, CA 92195-3308, USA (catalog # MBS2600246), represented the analytical tools used for measurement of the hepatic tissue content of SIRT1. Elabscience, Houston, TX, 77079, USA, was the source of the sandwich ELISA kits used for assessment of the hepatic tissue content of PPAR-γ (catalog # E-EL-R0724).

#### 4.7.3. Determination of the Hepatic Tissue Content of Kelch-like ECH-Associated Protein 1 (KEAP1), Nuclear Factor Erythroid 2-Related Factor 2 (Nrf2), and Heme Oxygenase-1 (HO-1)

Reddot Biotech, Katy, TX 77494, USA, provided us with the ELISA kits (Catalog # RD-KEAP1-Ra) used for the quantification of KEAP1 levels in the hepatic tissues. Nrf2 and HO-1 content of the hepatic tissues was determined using sandwich ELISA kits supplied by AFG Scientific, Northbrook, IL 60062, USA (SKU # EK720003 and EK240990, respectively).

#### 4.7.4. Quantification of the Hepatic Tissue Content of Interleukin 1-β (IL-1β), IL-6, Monocyte Chemoattractant Protein 1 (MCP-1), and Tumor Necrosis Factor Alpha (TNF-α)

Creative Biolabs, Shirley, NY 11967, USA, was the supplier of the kits used for the assessment of the hepatic tissue content of IL-1β and IL-6 (catalog # NHFF-0524-HX246 and NPP2011ZP228, respectively). MCP-1 levels in the hepatic tissues were quantified using kits purchased from BD Biosciences, San Jose, CA, USA (catalog # 555130). ELK Biotechnology Co., Denver, CO 80202, USA, was the provider of the ELISA kits used for the assay of the hepatic tissue content of TNF-α (catalog # ELK1396). The instructions enclosed within each of the aforementioned kits were the guide for the quantification of each of these parameters.

#### 4.7.5. Evaluation of HMGB1/RAGE/Nuclear Factor Kappa B (NF-κB) Axis in the Hepatic Tissues

Assay Genie Ltd., Dublin 2, Ireland, was the source of ELISA kits used for the determination of HMGB1 levels in the hepatic tissues (catalog # RTF100183). The hepatic tissue content of RAGE was quantified using the kits purchased from Creative Biolabs, Shirley, NY 11967, USA (catalog # NPP2011ZP428). NF-κB p65 levels in the hepatic tissue specimens were measured using the sandwich ELISA kits obtained from Sunlong Medical, Hangzhou, Zhejiang, China (catalog # NSL1325r).

#### 4.7.6. Assay of the Hepatic Tissue Levels of Phosphorylated Mammalian Target of Rapamycin (Phospho-mTOR), Total Adenosine Monophosphate-Activated Protein Kinase (AMPK), and LC3-II

The hepatic tissue content of phospho-mTOR (Ser2448) was determined using kits purchased from Cell Signaling Technology, Danvers, MA 01923, USA (catalog # 7976). Krishgen BioSystems, Worli, Mumbai 400018, India, was the provider of ELISA kits used for the assessment of the total AMPK in the hepatic tissues (catalog # KLR2094). LC3-II levels in the hepatic tissues were determined using kits supplied by Hoelzel Diagnostika Handels GmbH, Cologne, Germany (Item number YLA1417RA).

#### 4.7.7. Assessment of the Hepatic Tissue Content of Hydroxyproline, Matrix Metalloproteinase-3 (MMP-3), and MMP-9

Assay Genie Ltd., Dublin 2, Ireland, was the supplier of sandwich ELISA kits used for the quantification of the hepatic tissue content of hydroxyproline (catalog # RTEB1742). FineTest, Wuhan, 430074, Hubei, China, was the provider of ELISA kits used for the assay of MMP-3 and MMP-9 levels in the hepatic tissues (catalog # ER1164 and ER0139, respectively).

### 4.8. Light Microscopic Evaluation of the Morphologic Changes Induced by the Different Treatments in the Hepatic Tissue Specimens

After excision of the hepatic tissues, parts of these tissues were kept in 10% formaldehyde solution for 24 hrs at room temperature. After that, these tissues were dehydrated in ethanol and then embedded in paraffin to form paraffin blocks which were sliced at a 5 µm thickness using American Optical 820 microtome (American Optical Co., Vernon Hills, IL, USA). Then, these slices were put on glass slides where the hematoxylin and eosin (H&E) stain was applied. The stained sections were microscopically examined using a light microscope (AmScope Microscope and Accessories, Irvine, CA, USA). The histopathological changes in the hepatic tissues in the different groups were quantified using ImageJ software (Version 1.52f, National Institutes of Health, Bethesda, MD, USA).

### 4.9. Evaluation of the Immunohistochemical Expression of Cleaved Caspase 3 in the Hepatic Tissue Specimens of the Different Studied Groups

The immunohistochemical expression of cleaved caspase 3 was determined in formalin-fixed paraffin-embedded slices from the hepatic tissues as indicated by Eckle et al. [78]. Briefly, the formalin-fixed paraffin-embedded liver tissue samples were cut into thin sections (about 4–5 µm thick) by Leica EM UC6 ultramicrotome (Leica, Wetzlar, Hesse, Germany) and then deparaffinized using xylene and rehydrated through a series of graded alcohols to water. Antigen retrieval was performed to unmask the epitopes via using a microwave in a citrate buffer (pH 6.0). After that, the endogenous peroxidase activity was blocked by incubating the ultrathin tissue slices with hydrogen peroxide. Also, a blocking serum was utilized for blocking any non-specific binding sites. Then, the tissue sections were incubated overnight at 4 °C with a primary rabbit monoclonal antibody specific to cleaved caspase 3 obtained from Cell Signaling Technology, Danvers, MA 01923, USA (catalog # 9664). After washing, the tissue sections were incubated with a biotinylated secondary antibody (MyBioSource, San Diego, CA, USA, catalog # MBS9610384) that binds to the primary antibody. Then, an avidin-biotin complex (ABC) method was utilized to visualize the antibody binding. After that, the tissue sections were counterstained with hematoxylin to visualize the nuclei, dehydrated using a series of ascending grades of ethanol, cleared in xylene, and mounted with a coverslip using a Permount mounting medium. Olympus light microscope (Tokyo, Japan) was used for the assessment of the intensity of the positively stained cells, which was evaluated as mild (+), moderate (++), and strong (+++). The percentage of the positive immune expression of cleaved caspase 3 in the hepatic tissues in the different groups was quantified using ImageJ software (Version 1.52f, National Institutes of Health, Bethesda, MD, USA).

### 4.10. Clarification of the Alterations Induced by the Different Treatments in the Electron Microscopic Picture of the Hepatic Tissues

Parts of the excised liver tissue specimens were immersed in a solution containing 2% paraformaldehyde and 2.5% glutaraldehyde in 0.1 M cacodylate buffer (pH 7.4). They were then fixed in an ice-cold fixative for 20 h and placed in a mixture of 1% OsO_4_ and 0.8% K_4_[Fe (CN)_6_]. Following this, the specimens were dehydrated through a graded series of ethanol concentrations and embedded in epoxy resin. Ultrathin sections of the hepatic tissues, approximately 60 nm thick, were obtained using an ultramicrotome and then underwent thorough examination with a transmission electron microscope (JEM-1200EX, Jeol, Japan).

### 4.11. Statistical Analysis

The statistical program by which the results of the current study were analyzed was GraphPad Prism, version 9 (GraphPad Software, LLC, San Diego, CA, USA) where the obtained results were designated as the mean ± standard deviation (S.D.). The normal distribution of the results was assessed using the Shapiro–Wilk normality test followed by Bartlett’s test to determine the homogeneity of variances. The information obtained from the groups with equal variances was compared to each other by one-way analysis of variance (One-way ANOVA) after which a post hoc Tukey test was applied. Welch’s ANOVA test was utilized for the comparison between the groups with unequal variances followed by the Games Howell test. The determining point of statistical significance was set to be at a *p*-value under 0.05.

## 5. Conclusions

This study uncovered the potential role that may be played by perindopril in the mitigation of the hepatotoxic effects that occur as a result of treatment with methotrexate. This may be attributed to the ability of perindopril to modulate the key signaling pathways involved in the pathogenesis of this health problem including HMGB1/RAGE/NF-κB pathway, KEAP1/Nrf2/HO-1 signaling, SIRT1/PPAR-γ axis, and AMPK/mTOR-mediated autophagy. Also, further experiments are vitally needed to explore and identify the exact molecular mechanisms by which the different doses of perindopril may combat methotrexate-induced hepatotoxicity, including Western blotting and mRNA measurements of the target proteins in addition to experiments on knock-out rats. Moreover, further research efforts should be performed to give a fair evaluation of the possibility of introducing the findings of the current research to clinical settings.

## Data Availability

The original contributions presented in this study are included in the article/Supplementary Material. Further inquiries can be directed to the corresponding author.

## Supplementary Materials

The following supporting information can be downloaded at: https://www.mdpi.com/article/10.3390/ph18030358/s1.
